# Hands-On Robotic Microsurgery: Robotic-Assisted Free Flap Reconstruction of the Upper Extremity

**DOI:** 10.3390/jcm13237450

**Published:** 2024-12-06

**Authors:** Felix Struebing, Amir Khosrow Bigdeli, Arne Boecker, Jonathan Weigel, Ulrich Kneser, Emre Gazyakan

**Affiliations:** BG Trauma Center Ludwigshafen, Department of Hand, Plastic and Reconstructive Surgery, Heidelberg University, Ludwig-Guttmann-Straße 13, 67071 Ludwigshafen, Germany; felix.struebing@bgu-ludwigshafen.de (F.S.); arnehendrik.boecker@bgu-ludwigshafen.de (A.B.); ulrich.kneser@bgu-ludwigshafen.de (U.K.)

**Keywords:** robotic microsurgery, reconstructive microsurgery, robotic-assisted microsurgery, free flap reconstruction, upper extremity

## Abstract

**Background/Objectives**: Robot-assisted microsurgery (RAMS) has been introduced into the field of plastic surgery in recent years. It potentially offers enhanced precision and control compared to traditional methods, which is crucial for complex microvascular tasks in free flap reconstructions. We aim to analyze our experiences with robotic-assisted microsurgery in the field of upper extremity free flap reconstruction. **Methods**: This prospective study evaluated the efficacy and safety of the Symani Surgical System for free flap reconstructions in 16 patients with upper extremity defects at our institution from February 2023 to March 2024. Operating times were compared to a matched, historical cohort. We collected data on surgical outcomes, operative times, and complication rates, following strict adherence to the Declaration of Helsinki. **Results**: Our cohort primarily involved male patients (81%) with defects mostly located on the hand (81%). The anterolateral thigh flap was the most commonly used free flap (14/16, 88%). The average operative time was 368 ± 89 min (range: 216–550 min). No complete or partial flap losses were observed, but one flap required revision surgery due to arterial thrombosis. Major complications occurred in 13% of the cases. The average anastomosis time was 31 ± 12 min (range: 20–35 min) for arterial end-to-end anastomoses and 33 ± 13 min (range: 20–60 min) for arterial end-to-side anastomoses. Venous anastomoses required, on average, 20 ± 6 min. Operating times were not significantly longer when compared to the historical cohort (*p* = 0.67). **Conclusions**: We were able to show comparable outcomes to conventional microsurgery, while requiring more time for the microsurgical anastomoses. The study highlights the need for larger, controlled trials to better understand the benefits and limitations of robotic assistance in microsurgical reconstruction of the upper extremity.

## 1. Introduction

Managing traumatic soft tissue defects in the upper extremity, particularly in the hand, poses a significant challenge in reconstructive microsurgery. These defects often result from high-energy trauma, industrial accidents, military combat, or surgical resections, leading to significant functional impairment, aesthetic concerns, and a diminished quality of life for affected individuals [1,2]. Effective management requires a multidisciplinary approach, involving plastic surgeons, orthopedic surgeons, and hand therapists to optimize functional and aesthetic outcomes [3].

Robot-assisted microsurgery (RAMS) represents a paradigm shift in plastic surgery, offering enhanced precision, stability, and dexterity compared to traditional methods [4,5]. Robotic systems have already proven pivotal in several medical fields, such as urology and gynecology, where they facilitate meticulous dissection and suturing in confined spaces [6]. Applying these technological advancements to plastic surgery, especially in microsurgical procedures, holds the potential to improve outcomes by overcoming the inherent limitations of human hands and conventional instruments.

One of the most promising applications of robotic surgery in reconstructive microsurgery is free flap surgery [7]. Free flaps are a cornerstone in reconstructing complex soft tissue defects and require exceptional precision in microvascular anastomosis to ensure the viability of the transferred tissue. The ability of robotic systems to scale movements and filter physiological tremors may enhance the surgeon’s capability to perform delicate vascular anastomoses, which is paramount for the success of free flap reconstructions.

The Symani Surgical System (Medical Microinstruments, Jacksonville, FL, USA) offers features like motion scaling, tremor elimination, and seven degrees of freedom, making it particularly well suited for free flap reconstructions in the upper extremity. As we integrate advanced robotic systems like the Symani Surgical System into clinical practice, it is crucial to critically evaluate their impact on surgical outcomes, patient satisfaction, and advancements in reconstructive microsurgery. This study adds to the growing body of evidence supporting the use of robotic-assisted technologies in microsurgical practice, highlighting the potential benefits and challenges, and paving the way for future innovations in the field.

In this manuscript, we present a comprehensive study on the application of the Symani Surgical System in robotic-assisted microsurgical reconstruction of the upper extremity at our institution. This study aims to evaluate the efficacy, safety, and potential advantages of robotic-assisted microsurgery over traditional techniques in this particularly demanding patient cohort. By analyzing clinical outcomes, operative times, and complication rates, we seek to provide a critical assessment of the role and impact of robotic technologies, thus enhancing the standards of care for patients requiring complex reconstructive procedures.

## 2. Materials and Methods

### 2.1. Study Design

In February 2023, the Symani Surgical System (Medical Microinstruments, Jacksonville, FL, USA) was introduced to our institution and positioned in a designated operating room. All patients undergoing microsurgical interventions scheduled in this operating room were treated with robotic assistance. All cases were documented in a prospective database. This study analyzes all cases of robotic-assisted microsurgical soft tissue reconstruction of the upper extremity from February 2023 until 2024.

This study complies with the Declaration of Helsinki and was approved by the local ethics committee (Medical Commission Rhineland-Palatinate, Mainz, Germany; Approval number: 2023-16997).

### 2.2. Data Collection

We maintained a prospective database of all cases of robotic-assisted microsurgery in our department. Patient demographics (age, sex, body mass index (BMI), comorbidities, and risk factors) and surgical data (type of surgery, type of microsurgical reconstruction, operating time, duration of anastomosis, suture material used, number of stitches, surgical outcomes, and intra- and postoperative complications) were recorded. Data management was performed using Microsoft Excel (version 16.91; Microsoft Corporation, Redmond, WA, USA). In accordance with institutional policies, all data were collected in a pseudonymized form and stored on an encrypted and password-protected computer.

A comparison to a matched historical cohort of cases performed using the conventional technique during the timeframe of 2010–2018 was conducted to evaluate operating times between conventional cases and RAMS cases. The matching criteria included gender, age, type of flap, and location on the upper extremity. In one instance, an age mismatch was accepted due to the absence of a suitable matching case.

### 2.3. Surgical Technique

All free flap reconstructions were performed using standard free flap-raising techniques as previously described [8]. In each case, at least one anastomosis was performed using the Symani Surgical System. Standard robotic microinstruments were used throughout all cases. Optical magnification was achieved using either a conventional microscope (Mitaka MM51, Mitaka Kohki Ltd., Tokyo, Japan) or a digital exoscope with two large 4K-3D screens (Olympus OrbEye, Olympus K.K., Tokyo, Japan).

A total of seven surgeons performed the operations. Of these, two surgeons performed multiple reconstructions, with one surgeon completing seven cases and the other four, while the remaining surgeons performed one case each. All surgeons were skilled microsurgeons, each having performed more than one-hundred free flaps prior to the presented cases.

All surgeons underwent a comprehensive preclinical training program to ensure proficiency in controlling the robotic system. During this training, each surgeon was required to perform at least ten anastomoses of varying sizes (2.0–0.5 mm vessel diameter). Furthermore, end-to-end, end-to-side, and size-mismatched anastomoses were practiced. Using a three-dimensional model, microvascular anastomoses were simulated in a deep surgical field with limited access.

### 2.4. Statistical Analysis

Continuous variables are presented with means and standard deviation (SD) and discrete variables with median and interquartile range (IQR). Categorical variables are displayed with frequencies and percentages. Normality was evaluated using the Shapiro–Wilk test. Student’s t-test was used for the analysis of normally distributed data. The Mann–Whitney-U test was used for non-normally distributed data. Categorical data were analyzed using the chi^2^ test. Statistical significance was defined as *p* < 0.05.

All data were analyzed and visualized using GraphPad Prism (Version 10.1.1, GraphPad Software, San Diego, CA, USA).

## 3. Results

### 3.1. Patient Cohort

From February 2023 to March 2024, a total of 16 patients underwent robotic-assisted free flap reconstruction of the upper extremity at our institution. The mean age of the cohort was 54 ± 15 years, with the majority being male (14/16; 87.5%). The median American Society of Anaesthesiologists (ASA) classification was two with an interquartile range of one. The most frequent risk factor was arterial hypertension (6/16; 37.5%), followed by tobacco use (3/16; 18.8%) and diabetes (2/16; 12.5%). Table 1 depicts an overview of the patient characteristics.

The majority of defects affected the hand (13/16; 81.3%), while only two wounds were confined to the forearm (12.5%) and one to the elbow (6.3%). In six cases (37.5%), multiple anatomical regions, including the hand, forearm, elbow, and upper arm, were involved.

**Figure 1:**
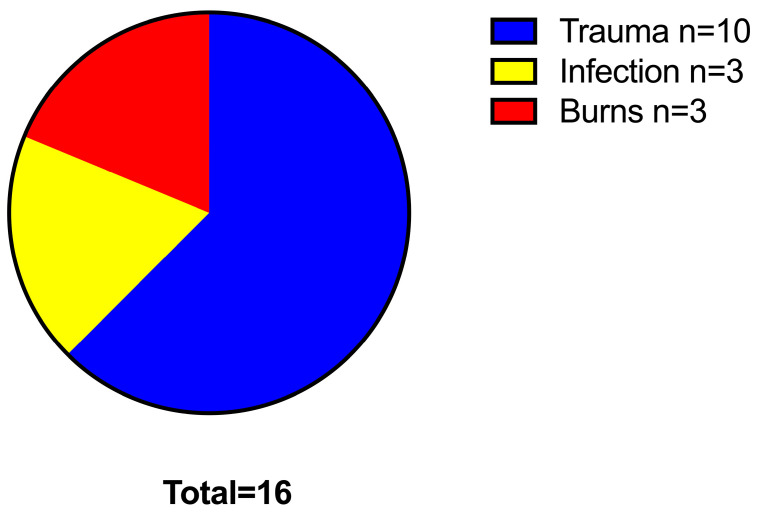
Pie chart showing the indications for the required free flap reconstructions.

Trauma was the leading cause of soft tissue defects (10/16; 63%), followed by infection (3/16; 19%) and burn injury (3/16; 19%). Figure 1 shows an overview of the indications indicating free flap reconstruction.

A historical comparison cohort (*n* = 16) was created to analyze whether the adoption of RAMS affected operating times. A matched-pair group was formed using the criteria of gender, age, type of flap, and flap location. No statistically significant differences were observed in age, gender, ASA classification, or comorbidities (all *p* > 0.05).

### 3.2. Operative Details

The most frequently used free flap was the anterolateral thigh flap, employed in fourteen cases (88%), followed by one medial femoral condyle flap (6%) and one latissimus dorsi free flap (6%). Recipient arteries included the radial artery in twelve cases (75%), the brachial artery in three cases (19%), and the ulnar artery in one case (6%). The average defect size measured 172 ± 121 cm^2^ (range 44–350 cm^2^), while the average flap size was 195 ± 120 cm^2^ (range 50–409 cm^2^).

The mean operating time was 368 ± 89 min (range: 216–550 min). A total of 30 robot-assisted anastomoses were performed across 16 cases. The largest group was the venous anastomoses with 17 (56.7%) end-to-end anastomoses. Arterial anastomoses were performed in eleven (36.7%) cases, including four end-to-end anastomoses and seven end-to-side anastomoses. There were three epineural coaptations (10%). The distribution of cases between the conventional optical microscope and digital exoscope was equal, with each modality used in half of the surgeries.

The operating time in the historical cohort of 16 patients, performed in the conventional hand-sewn technique, was 353 ± 109 min (range: 189–577 min). Operating times did not differ significantly between the two groups (*p* = 0.67).

The average time required for a robotic-assisted arterial anastomosis was 31 ± 12 min (range 20–60 min), with the end-to-side anastomoses taking the most time with 33 ± 13 min (range 20–60 min) and the end-to-end anastomosis with 26 ± 8 min (range 20–35 min). The completion of a venous anastomosis was achieved, on average, in 20 ± 6 min (range 11–34 min). The average duration of an epineural coaptation was 6 ± 1 min (range 5–7 min).

The median number of stitches for arterial end-to-side anastomoses was 11 (IQR 3), that for arterial end-to-end anastomoses was 6.5 (IQR 4), that for venous anastomoses was 7 (IQR 1.5), and that for epineural coaptations was 3 (IQR 0).

**Figure 2:**
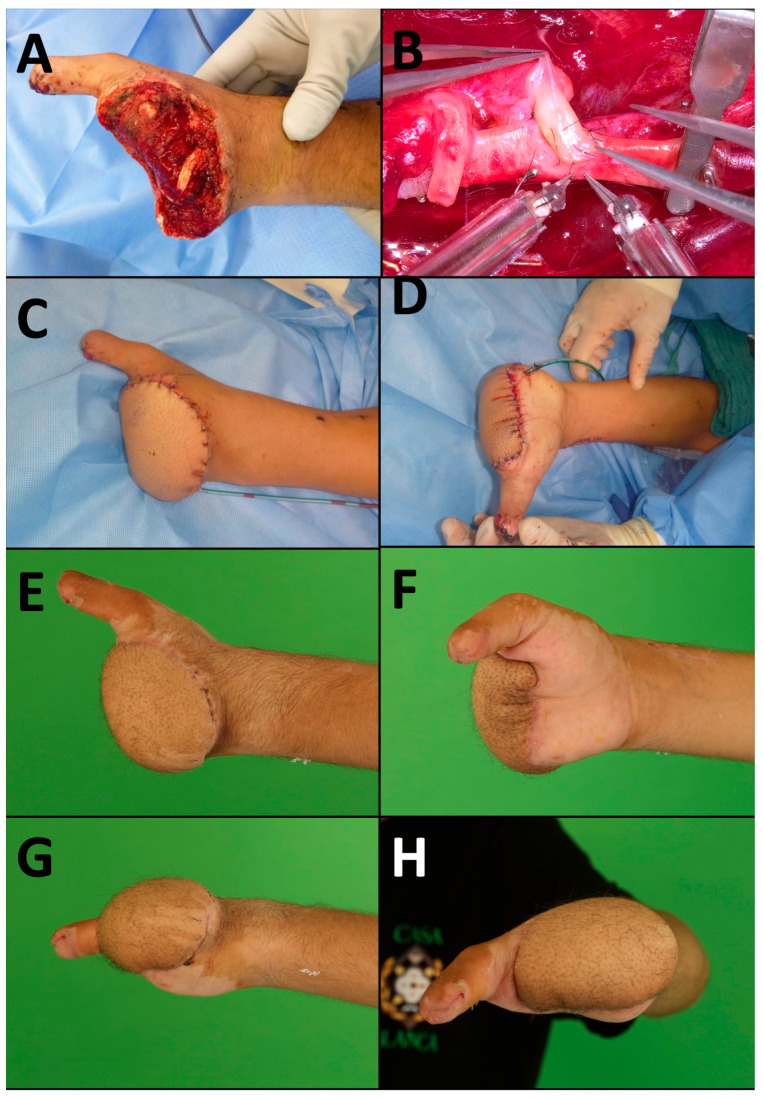
Exemplary case of a neurotized ALT reconstruction of the left hand of a young worker who had his left hand crushed by an excavator at the metacarpal level. Previously, a failed attempt at replantation had been undertaken at an external hospital. To reconstruct the remaining soft tissue defect, a free, neurotized ALT flap was raised and anastomosed to the radial artery. (**A**) Preoperative view of the soft tissue defect. (**B**) Vessel anastomosis using the Symani Surgical System. (**C**,**D**) The ALT flap is well perfused after inset. (**E**–**H**) Six weeks postoperatively, the flap was fully healed.

Regarding suture material, most venous anastomoses were performed using 9-0 sutures (9/17; 53%), followed by 8-0 sutures in seven cases (7/17; 41%) and 10-0 sutures in one case (1/17; 6%). Arterial end-to-end anastomoses were completed with 9-0 sutures in two cases (50%) and 10-0 sutures in the other two cases (50%). For arterial end-to-side anastomoses, 8-0 sutures were used in five cases (71%), and 9-0 sutures in the remaining two cases (29%). All epineural coaptations were performed using 9-0 sutures. Figure 2 shows an exemplary case of an ALT flap.

### 3.3. Complication Rate

Among the 16 free flaps performed, there were no instances of complete or partial flap loss. However, in one case involving a reconstruction of a large elbow defect with an ALT flap, the brachial artery developed an arterial thrombosis on the first postoperative day, necessitating an immediate return to the operating theater. Flap salvage was achieved by resection of the arterial anastomosis, thrombectomy, and a new hand-sewn arterial anastomosis.

Major complications occurred in two cases (13%). In addition to the aforementioned arterial thrombosis, there was one case of an incomplete healing of the skin-grafted ALT donor site, which required debridement and subsequent split-thickness skin grafting.

## 4. Discussion

This study evaluates the application of robot-assisted microsurgery (RAMS) in upper extremity defect reconstruction. The aim was to analyze its usability and the outcome in our daily microsurgical practice. Over the course of 13 months, we performed 16 consecutive robotic-assisted free flap reconstructions. We experienced neither complete nor partial free flap losses. Major complications were encountered in 13% of our cases. Our findings align with the current literature of soft tissue reconstruction of the upper extremity. Zhang and colleagues conducted a meta-analysis including 282 free flap reconstructions and reported a complete flap loss rate of 6.0% and a partial flap loss rate of 8.0% [9]. In a retrospective review of 282 cases of traumatic upper extremity reconstructions, Lakhiani et al. displayed a complete and partial free flap loss rate of 4.3% and 1.8%, respectively [10]. Similarly, Chang et al. reported a flap loss rate of 3.2% in upper extremity reconstructions [11].

The major complication rate of 13% is also consistent with published data. Wright et al. found a major complication rate of 9.9% in an NSQIP database study with 151 free flaps [12] in a retrospective analysis of 149 patients undergoing microsurgical reconstruction of the upper extremity.

Other teams have successfully utilized the Symani Surgical System in free flap reconstructions and reported results comparable to our study. Beier et al. reported a case series of 23 free flaps with 1 complete flap loss (4.3%) [13]. Innocenti et al. documented the first free flap reconstruction to be performed using the Symani robotic system [14]. Lindenblatt and colleagues presented a series of two vascularized lymph node transfers [5], while Barbon et al., from the same group, described three free flap reconstructions [15]. In both studies, no flap losses were encountered. In a retrospective study, Wessel et al. identified 28 patients who had undergone robot-assisted autologous breast reconstruction [16]. Mori et al. reported their findings of 16 free flaps, encountering no major complications [17].

It is important to note that robotic-assisted microsurgery tends to prolong anastomosis times due to factors such as motion scaling, draping, and the management of the robotic system. These findings are consistent with reports from other institutions using the Symani surgical system [4,13,18]. In the experimental setting, conventional vessel anastomosis of approximately 1 mm in diameter took between 13 and 23 min [19,20]. In our study, we reported anastomosis times ranging between 20 and 31 min using the robotic system, depending on the anastomosis type. Wessel et al. reported a comparison of two suture techniques using the Symani Surgical System and found that knot tying without switching the suture between needle holder and micro forceps was faster than switching sides after each individual knot [21]. Establishing standardized practices in robot-assisted microsurgery may further improve operating times and outcomes. Interestingly, we did not find the operating times to be significantly longer when comparing the presented cohort to a matched, historical cohort that had been performed in the conventional technique. The standardization of microsurgical practice may have contributed to shorter operating times, potentially compensating for the longer anastomotic durations.

Several authors have reported a relatively steep learning curve for robot-assisted microvascular anastomosis in the preclinical and clinical setting [15,16,22]. In our experience, however, this steep learning curve flattens significantly when transitioning from the pre-clinical to the clinical setting, and the rate of technical progression and speed improvement appear to decrease. This was especially true for microsurgeons with a comparatively low individual exposure to RAMS, characterized by fewer cases and longer intervals between them. We therefore hypothesize that a high case load is necessary for the individual microsurgeon to maintain optimal performance [18]. The plateau observed in the clinical learning curve may have several explanations: First, most microsurgeons performing RAMS are already highly experienced and, after a brief introduction to the technical aspects of the robotic system, quickly achieve a high level of performance, from which further progression is more challenging to demonstrate. Second, transitioning from controlled preclinical environments—where procedures are standardized—to clinical settings introduces variability in patient anatomy and surgical site complexity, which may pose additional challenges. Finally, as noted above, RAMS may require a consistently high case load to sustain optimal performance.

One significant hurdle to the broader adoption of robotic microsurgery is the cost, including high initial investment and the ongoing need for expensive single-use instruments. These costs are currently not being reimbursed in the German health care system and may impede further adoption even into the international market. The use of single-use instruments also raises concern in the context of climate change and the push towards increased sustainability in health care.

While our study demonstrates the feasibility of robotic-assisted microsurgery in upper extremity reconstruction, it is not without limitations. A major constraint is the absence of a control group, which limits our ability to draw definitive causal conclusions regarding the efficacy and safety of the robotic approach compared to traditional techniques. This limitation highlights the need for future randomized controlled trials to more accurately assess these outcomes. Additionally, despite our study being the largest known case series in this field, the relatively small sample size may reduce the statistical power of our analysis. The small cohort size restricts the generalizability of our findings to all patients undergoing similar procedures, as it may not fully capture the diversity of patient anatomies and surgical complexities encountered in broader clinical practice. Furthermore, not all cases were performed by the same surgeon, which could have introduced a performance bias. Thus, while our research provides valuable preliminary data, larger-scale studies are necessary to validate and refine the advantages and limitations of robotic-assisted techniques. The impact of robot-assisted microsurgery on surgeon health is another noteworthy aspect that warrants further investigation. Microsurgeons are subject to high musculoskeletal strain [23], which might be reduced by using robotic assistance and digital exoscopes.

## 5. Conclusions

The results of this study demonstrate that robot-assisted soft tissue reconstruction of the upper extremity is feasible. Preliminary results in a cohort of 16 patients undergoing free flap reconstruction at a single institution revealed no complete or partial flap losses. Moving forward, randomized, prospective studies are necessary to establish the non-inferiority or superiority of robot-assisted microsurgery in the reconstruction of the upper extremity.

## Figures and Tables

**Table 1:** Patient characteristics.

Parameter	Patient Cohort
Age, in years mean ± SD	54 ± 15
Male gender, *n* (%)	13 (81%)
Median ASA classification, median ± IQR	2 ± 1
**Comorbidities**	**Patient cohort *n*, (%)**
Arterial hypertension	6 (38%)
Tobacco use	3 (19%)
Diabetes	2 (13%)
PAOD	1 (6%)
Obesity	1 (6%)

SD: standard deviation; IQR: interquartile range; PAOD: peripheral arterial occlusive disease. Obesity was defined as BMI > 30 kg/m^2^.

## Data Availability

The data presented in this study are available on request from the corresponding author.
